# Editorial

**DOI:** 10.1120/jacmp.v8i3.2751

**Published:** 2007-08-20

The Canadian Organization of Medical Physicists (COMP) is the newest co‐sponsor of the JACMP, and that is excellent news, indeed. Canadian physicists have long been submitting to the JACMP. In fact some of our most excellent articles over the years had their origin at one of the significant Canadian institutions. As you know, the PKP software that supports the JACMP originates in Canada, and our publishing partner, Multimed, is a Canadian corporation. JACMP obviously owes a lot to our friends to the north, and I am personally very pleased that COMP is joining as a co‐sponsor.

I want to welcome Nathan Childress, Jianrong Dai, Chester Ramsey, and Frank Van den Heuvel to our Editorial Board as Associate Editors in Radiation Oncology Physics. Michael Schell joins as Associate Editor responsible for Book and Media reviews. Finally, George Starkschall joins as the new Editor‐in‐Chief for the JACMP, beginning with the Winter, 2008 issue. All that know him would agree that George is the perfect individual to take the JACMP to the next level. After 10 years with the JACMP, I will be moving on to another project. I will share more about that in the Fall, 2007 Editorial.

Michael D. Mills, PhD

**Table:** 

**THE JOURNAL OF APPLIED CLINICAL MEDICAL PHYSICS**	
**Editorial Board 2007**	
Editor‐in‐Chief	Michael Mills
Editor‐in‐Chief (beginning 2008)	George Starkschall
Deputy Editor‐in‐Chief	Timothy Solberg
**Associate Editors**	
Radiation Oncology Physics	Nzhde Agazaryan, Salahuddin Ahmad John Antolak, Nathan Childress Jianrong Dai, B. Gino Fallone Benjamin Nelms, Matthew Podgorsak Nikos Papanikolaou, Chester Ramsey William Salter, Mehrdad Sarfaraz Frank Van den Heuvel, Lu Wang Albert Zacarias
Medical Imaging	Geoffrey Clarke, William Pavlicek
Radiation Measurements	Larry DeWerd
Radiation Protection & Regulations	James Rodgers
Nonionizing Topics	Bhudatt Paliwal
Other Topics	Timothy Solberg
Book/Media Reviews	Michael Schell
Editorials	~
**Journal Production Team**	
Proofreader	Sheila Dietrich
Copy Editor	Ann Fothergill‐Brown
Layout Editor	Laura Shand
Manuscript Manager	Bonnie Ratcliff

